# A Mismatch-Tolerant Reverse Transcription Loop-Mediated Isothermal Amplification Method and Its Application on Simultaneous Detection of All Four Serotype of Dengue Viruses

**DOI:** 10.3389/fmicb.2019.01056

**Published:** 2019-05-08

**Authors:** Yi Zhou, Zhenzhou Wan, Shuting Yang, Yingxue Li, Min Li, Binghui Wang, Yihong Hu, Xueshan Xia, Xia Jin, Na Yu, Chiyu Zhang

**Affiliations:** ^1^School of Life Sciences, East China Normal University, Shanghai, China; ^2^Pathogen Discovery and Big Data Center, Chinese Academy of Sciences (CAS) Key Laboratory of Molecular Virology and Immunology, Institut Pasteur of Shanghai, Chinese Academy of Sciences, Shanghai, China; ^3^Medical Laboratory of Taizhou Fourth People’s Hospital, Taizhou, China; ^4^Faculty of Life Science and Technology, Kunming University of Science and Technology, Kunming, China; ^5^Viral Disease and Vaccine Translational Research Unit, CAS Key Laboratory of Molecular Virology and Immunology, Institut Pasteur of Shanghai, Chinese Academy of Sciences, Shanghai, China

**Keywords:** mismatch-tolerant LAMP, dengue virus, virus variant, high-fidelity DNA polymerase, mismatch, serotype

## Abstract

Loop-mediated isothermal amplification (LAMP) has been widely used in the detection of pathogens causing infectious diseases. However, mismatches between primers (especially in the 3′-end) and templates significantly reduced the amplification efficiency of LAMP, and limited its application to genetically diverse viruses. Here, we reported a novel mismatch-tolerant LAMP assay and its application in the detection of dengue viruses (DENV). The novel method features the addition of as little as 0.15 U of high-fidelity DNA polymerase to the standard 25 μl LAMP reaction mixture. This amount was sufficient to remove the mismatched bases at the 3′-end of primers, thereby resulting in excellent tolerance for various mismatches occurring at the 3′-end of the LAMP primers during amplification. This novel LAMP assay has a markedly improved amplification efficiency especially for the mutants forming mismatches with internal primers (FIP/BIP) and loop primers (FLP/BLP). The reaction time of the novel method was about 5.6–22.6 min faster than the conventional LAMP method regardless of the presence or absence of mismatches between primers and templates. Using the novel method, we improved a previously established pan-serotype assay for DENV, and demonstrated greater sensitivity for detection of four DENV serotypes than the previous one. The limit of detection (LOD) of the novel assay was 74, 252, 78, and 35 virus RNA copies per reaction for DENV-1, DENV-2, DENV-3, and DENV-4, respectively. Among 153 clinical samples from patients with suspected DENV infection, the novel assay detected 94.8% samples being DENV positive, higher than that detected by the commercial NS1 antigen assay (92.2%), laboratory-based RT-PCR method (78.4%), and the conventional RT-LAMP assay (86.9%). Furthermore, the novel RT-LAMP assay has been developed into a visual determination method by adding colorimetric dyes. Because of its simplicity, all LAMP-based diagnostic assays may be easily updated to the newly improved version. The novel mismatch-tolerant LAMP method represents a simple, sensitive and promising approach for molecular diagnosis of highly variable viruses, and it is especially suited for application in resource-limited settings.

## Introduction

Because of their high sensitivity and specificity, nucleic acid amplification (NAA) tests have been explored as molecular diagnosis tools for infectious diseases, especially for acute infections (Saijo et al., 2008; de Paz et al., 2014). There are two main types of NAA techniques: thermal cycling and isothermal amplification methods (de Paz et al., 2014). Thermal cycling amplification is the basis of various PCR-based techniques, such as the quantitative PCR (qPCR) method that has been widely applied to biomedical research, as well as agricultural, ecological, food and environmental sciences (Bustin, 2000, 2002; Saijo et al., 2008; Smith and Osborn, 2009; Kralik and Ricchi, 2017). In particular, a large number of commercial kits were developed using various qPCR techniques. However, PCR-based methods, especially qPCR, require relatively sophisticated equipment and highly trained personnel, to be performed in special diagnostic laboratories or facilities. These requirements limit their applications in resource-limited settings (Yang and Rothman, 2004).

Distinct from PCR-based methods, isothermal amplification is performed under a constant temperature, and rarely relies on sophisticated equipment (Yan et al., 2014; Zhao et al., 2015). Therefore, isothermal amplification techniques represent a promising direction for the development of point-of-care testing (POCT) method for use in the fields or resource-limited settings (de Paz et al., 2014). Some isothermal amplification methods have already been developed, such as nucleic acid sequence-based amplification (NASBA), loop mediated isothermal amplification (LAMP), rolling circle amplification (RCA), and recombinase polymerase amplification (RPA) (Yan et al., 2014; Zhao et al., 2015). Among them, LAMP is the most commonly used technique with more than 3,000 PubMed searchable publications by January, 2019 (Notomi et al., 2000). LAMP generally uses six primers to initiate the self-primed DNA synthesis and amplification cycling (Notomi et al., 2000; Nagamine et al., 2002). Although the most conserved genomic region is generally used for primer design, the LAMP primers, especially the two inner LAMP primers FIP and BIP that are typically over 40 nt long, form mismatches easily with templates of highly variable viruses that exist as a quasispecies (Sanjuan et al., 2010; Domingo and Perales, 2018). The mismatches between primers and templates can significantly reduce the amplification efficiency of LAMP (Notomi et al., 2000), decrease the sensitivity of detection, and even generate false negative results. These drawbacks are a major barrier to translate the LAMP technique into a commercial viable application (Wong et al., 2018), and the reason why few LAMP-based commercial diagnostic kits had been approved by the Food and Drug Administration (FDA) of US or World Health Organization (WHO) for the diagnosis of infectious diseases.

We recently developed a mismatch-tolerant RT-qPCR method with proof-reading capacity by introducing a small amount of high-fidelity DNA polymerase to a standard amount of Taq DNA polymerase (Li et al., 2019). The high-fidelity DNA polymerase removed the mismatched bases at 3′end of the primer and thus significantly improved the amplification efficiency for template containing mutations. Here, we utilized a similar principle to develop a mismatch-tolerant LAMP method for high-sensitive and broad-spectrum detection of genetically diverse viruses, and used dengue virus (DENV) in the proof-of-principle experiments.

DENV was chosen in part because it is a global public health problem with an estimated 390 million infections occurring each year (Bhatt et al., 2013). DENV is a positive-sense single-stranded RNA virus that belongs to the *Flaviviridae* family (Henchal and Putnak, 1990). Dengue epidemics occur mainly in urban and semi-urban areas in the tropical and subtropical regions, including a large number of resource-poor countries (Bhatt et al., 2013). The diseases caused by DENV infection vary from mild dengue fever to life-threatening dengue hemorrhagic fever or dengue shock syndrome (Henchal and Putnak, 1990; Gubler, 1998; Ligon, 2005). Symptomatic DENV infections are readily recognizable by clinical manifestation, serological and molecular diagnostic assays. However, the majority of individuals with DENV infection are subclinical, with no manifested symptoms, yet capable of spreading the virus (Bhatt et al., 2013). Therefore, early and accurate diagnosis of DENV infection, using a relatively simple method that can be deployed to different terrains, is critical not only to clinical management, but also for the prevention and control of dengue epidemic (Teles et al., 2005).

Part of the difficulty in dengue diagnosis is that DENV comprises four antigenically distinct serotypes of DENV-1, DENV-2, DENV-3, and DENV-4, and different serotypes only exhibit 65–70% sequence homology at the nucleotide level (Henchal and Putnak, 1990; Ligon, 2005). Although some detection assays, including antibody detection (IgM and IgG), antigen detection (non-structural protein 1, NS1), and various NAA assays for individual or multiple serotypes, had been developed (Shu and Huang, 2004; Teles et al., 2005), there still lacks a high-efficiency pan-serotype DENV detection assay for point-of-care use in resource-limited settings. Using the novel mismatch-tolerant LAMP technique, we improved the sensitivity of detection of a previous developed pan-serotype RT-LAMP assay for DENV, and demonstrated that such a test may have practical applications.

## Materials and Methods

### Viruses

Four DENV strains DENV-1 (16007), DENV-2 (16681), DENV-3 (16562), and DENV-4 (1036) were used to establish the novel DENV RT-LAMP assay. Two other flaviviruses, Zika virus (SZWIV-01) and attenuated yellow fever virus 17D (derived from Asibi strain), and eight respiratory viruses were used to evaluate the cross-reactivity of the assay. The respiratory viruses include adenovirus (VR-930), enterovirus (VR-1076), influenza A and B viruses (VR-333 and VR-789), parainfluenza viruses 3 (VR-93), HCoV-229E (VR-740), HCoV-OC43 (VR-1558), and human rhinovirus (VR-489). Four DENV strains were gifts from Dr. Claire Huang (U.S Centers for Disease Control and Prevention at Fort Collins, Colorado). The Asian ZIKV strain SZ-WIV01, isolated from human serum of an imported case of ZIKV infection in China in 2016, was obtained from Wuhan Institute of Virology, Chinese Academy of Sciences. The yellow fever virus 17D was obtained from Dr. Cheng-feng Qin (Academy of Military Medical Sciences, Beijing). The respiratory viruses were previously purchased from the American Type Culture Collection (ATCC) and stored at Institut Pasteur of Shanghai, Chinese Academy of Sciences.

### RNA Extraction

Viral RNA was extracted from 140 μl virus culture supernatants or patients’ plasma using the QIAamp Viral Min Kit (Qiagen, Germany). RNA was eluted in 50 μl nuclease-free water and stored at -80°C until use.

### Construction of Mutant Viral RNA Template

To perform the proof-of-concept experiment of the mismatch-tolerant RT-LAMP, a series of mutants that form mismatches with the F3, FIP and BLP primers were constructed using a fast mutagenesis system (TransGen, Beijing, China) based on the 3′-UTR region of the DENV-4 genome. In brief, the 3′-UTR fragment of DENV-4 was obtained using StarScript II Probe One-Step qRT-PCR Kit (GenStar, Beijing, China) with primers F3/134 and B3/4, and then sub-cloned into pMD18-T plasmid vector (TaKaRa, Dalian, China). A T7 promoter was fused to the 5′-end of the primer F3/134. Various mutants were constructed using site-directed mutagenesis with mutagenic primers (Supplementary Table S1) and confirmed by Sanger sequencing. Mutant RNAs were obtained through *in vitro* transcription with T7 RNA polymerase, and quantified using spectrophotometric absorbance at 260 nm (NanoDrop, Technologies Inc.). Copy number of each mutant RNA was calculated using the following formula: RNA copies/μl = [RNA concentration (g/μl)/(nt transcript length × 340)] × 6.022 × 10^23^.

### Reaction System of the Novel RT-LAMP Assay

Bst 2.0 DNA polymerase, WarmStart^®^ RT and Q5 High-Fidelity DNA polymerase (all from New England Biolabs, Beverly, MA, United States) were used to establish the novel mismatch-tolerant RT-LAMP system. The main difference between the novel and the conventional RT-LAMP assays is the inclusion of an additional amount of high-fidelity DNA polymerase in the reaction system. The amount of the Q5 high-fidelity DNA polymerase was optimized at 0.15 U per 25 μl reaction mix. A 25 μl reaction mix of the novel RT-LAMP assay includes 1× isothermal amplification buffer, 6 mM MgSO4, 1.4 mM dNTPs, 8 units of Bst 2.0 DNA polymerase, 7.5 units of WarmStart^®^ RT, 0.15 unit of Q5 High-Fidelity DNA Polymerase, 20 pmol each of primers FIP/123, FIP/4, BIP/123, and BIP/4, 2.5 pmol each of primers F3/134, F3/2, B3/123, and B3/4, and 20 pmol of loop primer BLP/1234. Three microliter of RNA was used for the RT-LAMP assays. The primer information was previously described and provided as Supplementary Table S1 (Teoh et al., 2013). The RT-LAMP reaction was performed at 63°C for 60 min with 0.4 μM SYTO 9 (Life technologies, Carlsbad, CA, United States) as fluorescent dye for real-time monitoring by the Light Cycler 96 real-time PCR System (Roche Diagnostics, Mannheim, Germany).

### Sensitivity and Limit of Detection (LOD)

Ten-fold serial dilution of the RNA from four DENV stocks were used as the standards to determine the sensitivity of the novel DENV RT-LAMP assay. The initial titers of the DENV-1, DENV-2, DENV-3, and DENV-4 stocks were 4 × 10^6^, 1 × 10^7^, 2 × 10^4^, and 1.6 × 10^7^ plaque-forming unit (PFU) per ml. To determine the RNA copy numbers of the virus stocks, RT-qPCR assay was performed with previously described primers (Supplementary Table S1; Go et al., 2016), and a standard curve was obtained by 10-fold serial dilutions of *in vitro* transcribed DENV RNA standard from 10^7^ to 10^2^ copies/μl. The RNA copy numbers of DENV-1, DENV-2, DENV-3, and DENV-4 stocks were quantified to be 1.6 × 10^8^, 1.1 × 10^8^, 9.3 × 10^6^, and 1.2 × 10^7^ copies/ml, respectively.

LOD of the novel DENV RT-LAMP assay was determined using 10-fold serial dilutions of RNA from each virus stock. Each dilution was tested in a set of 10 replicates. To more accurately estimate the LOD, we performed additional experiments using a fivefold dilution of each DENV serotype with a starting RNA copy number of 3 × 10^4^. Probit regression analysis was performed to determine the LOD using the SPSS 17.0 software. The LOD was defined as a 95% probability of obtaining a positive result (Anderson, 1989).

### Evaluation of the Novel DENV Detection Assay Using Clinical Samples

To evaluate the performance of the novel RT-LAMP assay for pan-serotype detection of DENV, 153 plasma samples that were previously collected from dengue-suspected patients (Wang et al., 2016, 2018). DENV infection was determined by both NS1 antigen detection and specific RT-PCR assays. The NS1 antigen assay was performed using One-Step Dengue NS1 RapiDip^TM^ InstaTest kit (Cortez Diagnostics, United States); the RT-PCR assay was performed using PrimeScript^TM^ One Step RT-PCR Kit Ver.2 (Takara, Japan). Of the 153 samples, 120 positive samples by RT-PCR assay were further subjected to Sanger sequencing and phylogenetic analysis, and 58 DENV-1, 5 DENV-2, 46 DENV-3, and 11 DENV-4 were identified. During September, 2018, the RNA stocks were tested again using both the novel and the conventional RT-LAMP assays. A comparison in performance of the four methods was performed, and the concordance rate was calculated by Clinical Calculator with the following formula: (number of consistent results by both methods/total number) × 100%^1^.

The samples having a negative result by RT-PCR assay but positive by the novel RT-LAMP assay, or having a time threshold (Tt) difference of more than 15 min between the novel and the conventional RT-LAMP assays were subjected to further RT-nested PCR amplifications. The amplicons were processed for Sanger sequencing and further sequence analyses. The RT-nested PCR assay was performed using StarScript II Probe One-Step qRT-PCR Kit (GenStar, Beijing, China) with F3 and B3 as outer primers, and F2 and B2 as inner primers (Supplementary Table S1).

### Visual Detection

To develop a visual detection method, 120 μM hydroxynaphthol blue (HNB) (Sigma-Aldrich, United States) was added to the novel and the conventional RT-LAMP reaction mix. Another visual RT-LAMP assay was performed with the WarmStart Colorimetric LAMP 2X Master Mix (New England Biolabs, Beverly, MA, United States), which uses cresol red as a visual indicator. The reactions were performed at 63°C, and the color change was observed at the 30-, 40-, 50-, and 60-min time points.

### Ethics Statement

This study was approved by the Ethics Committees of Shanghai Public Health Clinical Center (No. 2014-008) and Kunming University of Science and Technology (No. 2018JC027). Written informed consents were obtained from all dengue-suspected patients.

## Results

### The Establishment of a Mismatch-Tolerant RT-LAMP Assay

**Figure 1:**
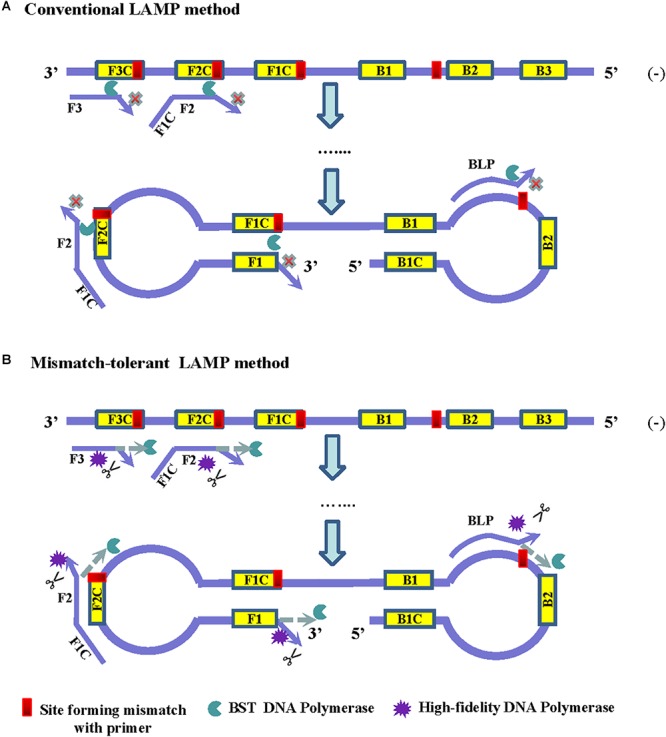
Principles of the conventional **(A)** and the mismatch-tolerant LAMP **(B)** methods. The presence of a mismatch at 3′-end of the primer will largely reduce or stop LAMP amplification. The presence of a small amount of high-fidelity DNA polymerase, which has 3′–5′ exonuclease activity, can remove the mismatched bases from the primers, allowing the Bst DNA polymerase to initiate primer extension.

The principle of LAMP shows that the 3′-ends of the F2/B2, F3/B3, and LF/LB, as well as the 3′-ends of F1/B1 (corresponding to the 5′-ends of the F1c/B1c) provide 3′-hydroxyl groups for DNA extension (Notomi et al., 2000). Because of lacking a 3′–5′ exonuclease activity, Bst2.0 DNA polymerase has a reduced capacity to extend primers when mismatches occur at the 3′-ends of these primers, and thus resulted in a low amplification efficiency of LAMP (Figure 1A). Removing the mismatched bases at the 3′-end of the primers by an additional enzyme can overcome the inhibition effect of mismatches on LAMP reaction.

High-fidelity DNA polymerase has a proofreading function and can be used to remove mismatched nucleotides. To improve the amplification efficiency of LAMP on highly variable templates, we developed a mismatch-tolerant LAMP method by combining Bst 2.0 DNA polymerase and a small amount of high-fidelity DNA polymerase in the reaction (Figure 1B). The amount of high-fidelity DNA polymerase was previously demonstrated to be optimal at 0.15 U in a 25 μl reaction system, at which the mismatched bases were removed from the primers, and the completion with other DNA polymerase such as Taq DNA polymerase for DNA synthesis did not occur (Hao et al., 2015; Li et al., 2019). The same amount of high-fidelity DNA polymerase was also demonstrated to be optimal for the mismatch-tolerant LAMP method (data not shown). Therefore, a standard mismatch-tolerant LAMP reaction system that includes all reagents in the conventional LAMP reaction with an additional 0.15 U high-fidelity DNA polymerase were established. Using the novel system, we developed an novel RT-LAMP assay for pan-serotype detection of DENV using the primer sets previously described (Teoh et al., 2013).

### Validation of the Mismatch-Tolerant RT-LAMP Assay

**Figure 2:**
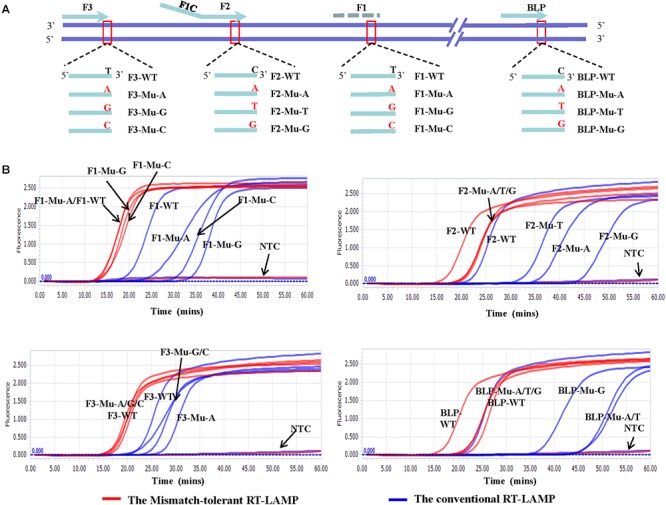
Flexibility of the novel mismatch-tolerant RT-LAMP to various mismatches. **(A)** Primer and template sets. **(B)** Amplification curves of the novel mismatch-tolerant and the conventional RT-LAMP methods for various mutants that form mismatches with the primers. About 30,000 RNA copies of mutant or wild-type template were added in each reaction. WT, wild-type; Mu, mutant; NTC, no template control.

To assess the influence of various mismatches between primers and templates on LAMP amplification, and validate the effectiveness of the novel mismatch-tolerant RT-LAMP, we constructed a series of mutant RNA of DENV-4 that can form three kinds of mismatches with the 3′-ends of F3, FIP, and BLP, as well as the 5′-end of FIP (i.e., 3′-end of F1) (Figure 2A). We firstly used the conventional RT-LAMP assay to detect 3 × 10^4^ copies of the mutant RNAs, and a control wild type template. Relative to the wild type template (Tt: 21.7 min), the mutants generated slower RT-LAMP amplification with Tt values of 22.8–44.3 min (Figure 2B and Table 1). For the same primer, three kinds of mismatches formed with the mutant RNAs resulted in a relatively large variance in Tt values (0.5–6.5 min) (Table 1). In particular, we found that the mismatches occurring in different primers had marked difference in their inhibition effect on LAMP amplification. The mutants forming mismatches with the 3′-end of the F3 primer had Tt values of 22.8–25.2 min (about 1–3.5 min higher than the wild type), whereas the mutants forming mismatches with the 3′-end of the BLP or FIP (corresponding to the 3′-end of F2) primers had Tt values of 35.2–44.3 min, which are 13.5–22.6 min higher than the wild type (Table 1).

**Table 1:** The influence of various mismatches between primers and templates on the amplification times by the novel mismatch-tolerant and the conventional RT-LAMP methods.

Template	The novel mismatch-tolerant RT-LAMP						The conventional RT-LAMP						Tt Diff. (Novel-Conv.)	
		
	Tt-T1 (min)	Tt-T2 (min)	Tt-T3 (min)	Mean	SD	Tt Diff. (Mut-WT)	Tt-T1 (min)	Tt-T2 (min)	Tt-T3 (min)	Mean	SD	Tt Diff. (Mut-WT)	
Wild	17.02	17.1	14.09	16.1	1.7	**NA**	22.1	22.9	20.0	21.7	1.5	**NA**	**-5.6**
F1-Mu-A	15.51	15.28	13.87	14.9	0.9	**-1.2**	26.5	33.8	25.7	28.6	4.5	**6.9**	**-13.7**
F1-Mu-G	17.22	16.4	14.86	16.2	1.2	**0.1**	33.5	37.1	34.7	35.1	1.8	**13.4**	**-18.9**
F1-Mu-C	16.82	17.43	14.61	16.3	1.5	**0.2**	31.8	33.5	31.3	32.2	1.1	**10.5**	**-15.9**
F2-Mu-A	21.1	21.73	18.14	20.3	1.9	**4.3**	36.4	38.9	30.3	35.2	4.4	**13.5**	**-14.9**
F2-Mu-T	20.64	22.52	18.56	20.6	2.0	**4.5**	32.8	41.1	34.3	36.1	4.4	**14.4**	**-15.5**
F2-Mu-G	20.73	20.82	19.78	20.4	0.6	**4.4**	44.9	36.7	35.3	39.0	5.2	**17.3**	**-18.5**
F3-Mu-A	17.55	15.93	15.21	16.2	1.2	**0.2**	27.9	25.3	21.0	24.7	3.5	**3.0**	**-8.5**
F3-Mu-G	16.93	17.57	16.35	17.0	0.6	**0.9**	23.0	23.7	21.7	22.8	1.0	**1.1**	**-5.9**
F3-Mu-C	18.09	16.66	15.44	16.7	1.3	**0.7**	25.4	28.1	22.1	25.2	3.0	**3.5**	**-8.4**
BLP-Mu-A	23.45	21.55	19.09	21.4	2.2	**5.3**	46.7	45.1	35.0	42.2	6.3	**20.6**	**-20.9**
BLP-Mu-T	21.81	24.04	19.48	21.8	2.3	**5.7**	46.8	49.7	36.6	44.3	6.9	**22.6**	**-22.6**
BLP-Mu-G	21.97	24.18	21.43	22.5	1.5	**6.5**	37.6	40.3	39.1	39.0	1.3	**17.3**	**-16.5**

*SD, standard deviation; NA, not applicable; Diff., difference; Mut, mutants; WT, wild type; Tt, time threshold; T1-T3, replicates 1–3; Conv., conventional RT-LAMP method; Novel, the mismatch-tolerant RT-LAMP method; min, minute. The Tt differences are highlighted in bold.*

Then, we used the mismatch-tolerant RT-LAMP assay to test the same mutants and the wild type template. The novel method had Tt value of 16.1 min for the wild type template, about 5.6 min lower than that in the conventional method (Figure 2B and Table 1). A dramatic improvement on the LAMP amplification was achieved by the novel method for all mutants (about 5.9–22.6 min faster than the conventional method) (*P* < 0.001, paired *T*-Test) (Table 1). The mutants forming mismatches at the 3-ends of both the BLP and FIP, as well as at the 5′-end of the FIP (corresponding to the 3′-end of F1) had about 13.7–22.6 min lower Tt values by the novel method than the conventional assay, and the mutants having mismatches with the F3 primer showed about 5.9–8.5 min lower Tt values than the conventional ones (Table 1). Importantly, the novel method seemed to have a similar amplification efficiency for the wild type and the mutants, except for slightly slower (about 4.3–6.5 min) for the mutants forming mismatches with the 3′-ends of the FIP and BLP than for the wild type (Table 1).

### Pan-Serotype Detection of DENV

**Figure 3:**
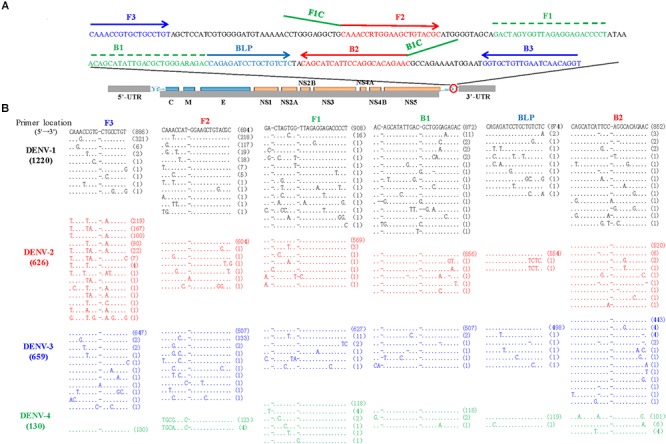
Locations **(A)** and sequence alignments **(B)** of the primer regions of all available DENV strains. A total of 1220 DENV-1 (black), 626 DENV-2 (red), 659 DENV-3 (Blue), and 130 DENV-4 (green) sequences were downloaded from GenBank on September 10, 2018. Dot, identity with the topmost sequence; Dash, deletion.

**Figure 4:**
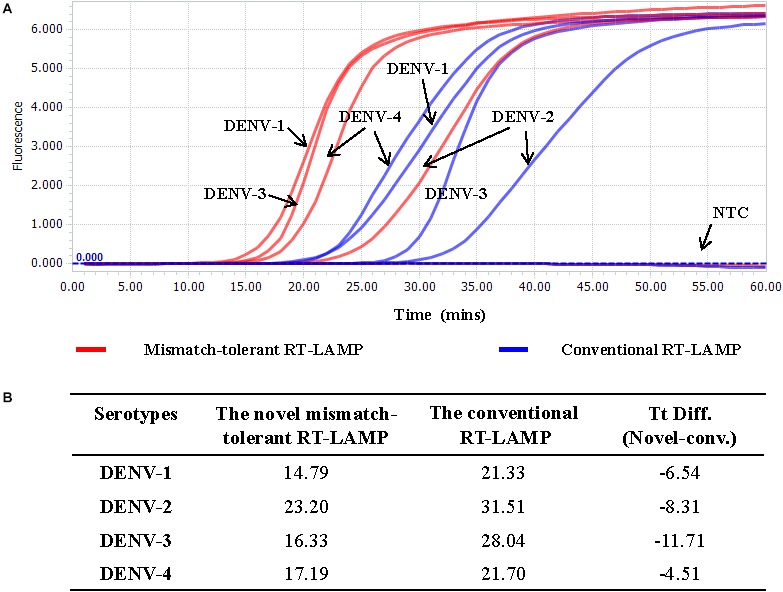
Detection of four DENV serotypes using the novel mismatch-tolerant and the conventional RT-LAMP assays. **(A)** Amplification curves of the novel and the conventional RT-LAMP methods. **(B)** Comparison of the Tt values between the novel and the conventional RT-LAMP. About 3,000 RNA copies of each DENV serotype was used in the test. Mins, minutes; NTC, no template control.

The LAMP primers for pan-serotype detection of DENV were previously designed in the 3′ UTR region of the DENV genome, the most conserved genomic region shared among the four serotypes (Figure 3A). To further evaluate the primers, we retrieved all available 3′ UTR sequences of four DENV serotypes, and performed sequence alignments. A total of 1220 DENV-1, 626 DENV-2, 659 DENV-3, and 130 DENV-4 sequences were obtained (Figure 3B). Except for the F3 region, the vast majority of sequences are conserved for all four serotypes, and only a few variants in each serotype can form mismatches with the LAMP primers (Figure 3B). Furthermore, DENV-2 and DENV-4 appeared to be distinct from DENV-1 and DENV-3 in the F3 and F2 regions (especially the regions corresponding to the 5′ parts of the primers) (Figure 3B). When all four DENV strains were detected using the conventional RT-LAMP assay, a lower amplification efficiency was observed for DENV-2 (Figure 4A). Sequencing and sequence analysis showed that there were four substitutions in the FIP and BIP regions of DENV-2 compared to other serotypes (Supplementary Figure S1). To examine whether the mismatch-tolerant RT-LAMP can improve the amplification efficiency for all four DENV serotypes, we used the novel method to detect the same DENV strains and compared it with the conventional RT-LAMP assay. All four serotypes had lower Tt values (14.8–23.2 min) by the novel RT-LAMP assay than that by the conventional ones (21.3–31.5 min) (Figure 4B). The differences in Tt values between the two assays were 4.5–11.7 min. To avoid non-specific amplification, the reaction time of the new method was set at 50 min for the subsequent experiments.

### Cross-Reactivity and Limit of Detection of the Novel RT-LAMP Assay

**Figure 5:**
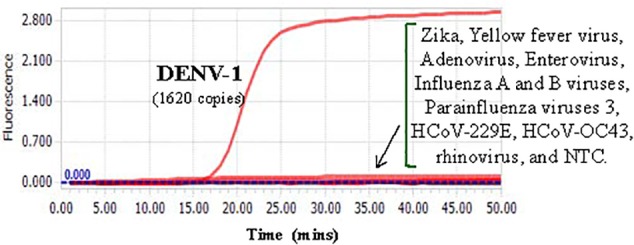
Cross-reactivity test of the novel DENV RT-LAMP assay to other human viruses. About 1620 RNA copies (33.6 PFU) of DENV-1 was used as the positive control. Testing viruses included Zika, Yellow fever virus, Adenovirus, Enterovirus, Influenza A and B viruses, Parainfluenza viruses 3, HCoV-229E, HCoV-OC43, and rhinovirus. NTC, no template control.

**Figure 6:**
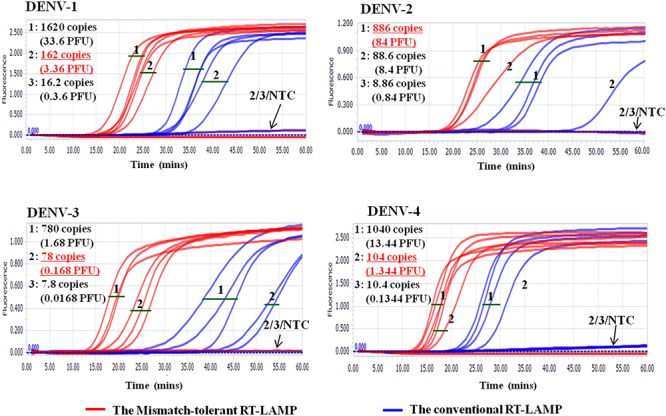
Sensitivity comparison between the novel mismatch-tolerant and the conventional RT-LAMP assays for four DENV serotypes. The sensitivity of the novel mismatch-tolerant RT-LAMP assay for each DENV serotype is highlighted in red and underline. Positive amplification was defined only when all three replicates are successfully amplified. NTC: no template control.

Cross-reactivity test showed that there was no amplification of other ten human viruses (including closely related flaviviruses, Zika virus, and yellow fever virus) by the novel DENV RT-LAMP assay within 50 min (Figure 5), indicating that the new assay is specific for DENV. Sensitivity tests showed that the novel pan-serotype DENV RT-LAMP can detect as low as 162, 886, 78, and 104 RNA copies of DENV-1, DENV-2, DENV-3, and DENV-4, which are equivalent to 3.36, 84, 0.17, and 1.34 PFU, respectively. In comparison, the conventional RT-LAMP assay only yielded one or two positive amplifications among the three replicates at the same concentrations of DENV-1, DENV-3 and DENV-4, except for DENV-2, for which 886 virus RNA copies were detected by all replicates (Figure 6). Of particular importance is that all the amplification curves by the novel RT-LAMP assay appeared within 25 min, whereas almost all curves by the conventional assay appeared after 25 min (Figure 6). The LOD values of the novel assay, as described in the materials and methods, were determined as 74, 252, 78, and 35 RNA copies per reaction for DENV-1, DENV-2, DENV-3, and DENV-4, respectively (Table 2).

**Table 2:** Limit of detection (LOD) of the novel mismatch-tolerant RT-LAMP.

Dilution	Standard (copies/reaction)	Positive/total tested			
		DENV-1	DENV-2	DENV-3	DENV-4
	30,000	10/10	10/10	10/10	10/10
5×	6000	10/10	10/10	10/10	10/10
5×	1200	10/10	10/10	10/10	10/10
5×	240	10/10	9/10	10/10	10/10
5×	48	4/10	0/10	8/10	10/10
5×	9.6	0/10	0/10	4/10	1/10
**LOD** (copies/reaction)		74	252	78	35

### Visual Detection

**Figure 7:**
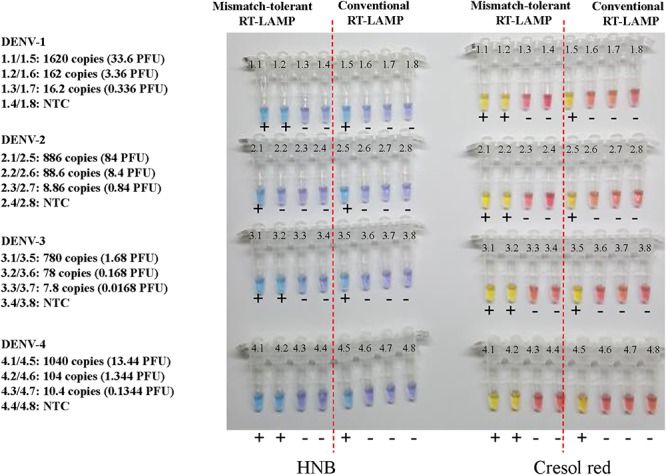
Visual detection of four DENV serotypes using the novel mismatch-tolerant and the conventional RT-LAMP assays with HNB or cresol red. The reactions were performed at 63° for 50 min. The color changes from violet to azure for HNB and from burgundy to orange or yellow for cresol red were considered as positive (+). The results at 30, 40, and 60 min are shown in Supplementary Figure S2.

To prepare for use in the resource-poor settings, the novel RT-LAMP assay was further developed into a visual determination assay by adding HNB or cresol red. Because both HNB and Cresol Red assays gave a clear color indication for all samples at 50 min (Figure 7 and Supplementary Figure S2), it was now selected as the cut-off for the visual assays. DENV-1, DENV-2, DENV-3, and DENV-4 were detected by the novel assay at concentrations of 162, 89, 78, and 104 copies, respectively (Figure 7), showing the same sensitivity as the novel RT-LAMP with real-time monitoring. As a comparison, the conventional assay only detected 1620, 886, 780, and 1040 copies of DENV-1, DENV-2, DENV-3, and DENV-4, respectively, for the same duration of amplification.

### Evaluation of the Novel Pan-Serotype DENV RT-LAMP Assay

To explore the potential for clinical application of our novel assay, a total of 153 plasma samples collected from dengue-suspected patients were used to evaluate the novel DENV RT-LAMP assay. Of these plasma samples, 106 (69.3%) were detected as DENV positive by all four assays (Supplementary Figure S3), and various proportions were detected as being positive by individual assay.

The novel RT-LAMP, the NS1 antigen detection, the specific RT-PCR and the conventional RT-LAMP assays detected 145 (94.8%), 141 (92.2%), 120 (78.4%), and 133 (86.9%) DENV positive samples, respectively (Table 3). The concordance rates of the novel RT-LAMP assay were 90.8% [95% confidence interval (CI): 84.8–94.7%; kappa value: 0.253] and 78.4% (95% CI: 70.9–84.5%; kappa value: 0.121) with the NS1 antigen detection and the specific RT-PCR assays, respectively (Table 3). The positivity results were more consistent between the novel and the conventional RT-LAMP assays, with a concordance rate of 92.2% (95% CI: 86.4–95.7%; kappa value: 0.537).

**Table 3:** Comparison among different DENV detection assays for 153 clinical samples.

Methods		NS1 antigen assay			RT-PCR			The conventional RT-LAMP			Total	Positive rate (%)
		
	Items	Pos.	Neg.	Concordance rate (%)	Pos.	Neg.	Concordance rate (%)	Pos.	Neg.	Concordance rate (%)		
The novel mismatch-	Pos.	136	9	90.8%	116	29	78.4%	133	12	92.2%	145	94.8%
tolerant RT-LAMP	Neg.	5	3		4	4		0	8		8	
Total		141	12	NA	120	33	NA	133	20	NA	153	NA
Positive rate (%)		92.2%		NA	78.4%		NA	86.9%		NA	NA	NA

*The concordance rate was calculated using the formula: (number of consistent results by both methods/total number) × 100%. Please also see the Venn diagram in Supplementary Figure S3 for details. Pos., positive; Neg., negative; NA, not applicable.*

To assess the capacity of the novel RT-LAMP assay for detection of all four DENV serotypes, 120 samples that were previously genotyped were used, and 116 of them (96.7%) were also tested as positive by the novel RT-LAMP assay (Table 4). Among them, all DENV-2 (5/5, 100%) and DENV-3 (46/46, 100%), most DENV-1 (55/58, 94.8%), and DENV-4 (10/11, 90.9%) samples were detected as being positive (Table 4). The novel RT-LAMP assay showed better performance for DENV-1 (94.8% vs. 87.9%) and DENV-3 (100% vs. 87.0%) than the conventional assay, and identical performance for DENV-2 and DENV-4 (Table 4). In addition, we randomly selected 12 samples with a negative RT-PCR test but positive by the novel RT-LAMP assay for further amplification using a RT-nested PCR assay. One additional sample was detected as being positive and further genotyped as DENV-4 by Sanger sequencing and Blast analysis. Among seven samples with a Tt difference of more than 15 min between the novel and the conventional RT-LAMP assays, three DENV-1 and four DENV-3 strains were identified, and only the three DENV-1 strains carried A- > C or C- > T substitution in the F2 region (Supplementary Figure S4). The two substitutions were also found in some previously sequenced DENV-1 variants (Figure 3).

**Table 4:** Detection rates of different DENV serotypes by the novel mismatch-tolerant and the conventional RT-LAMP assays.

DENV serotypes	The novel mismatch-tolerant RT-LAMP			The conventional RT-LAMP			Total
		
	Positive	Negative	Detection rate (%)	Positive	Negative	Detection rate (%)	
DENV-1	55	3	94.8	51	7	87.9	58
DENV-2	5	0	100	5	0	100	5
DENV-3	46	0	100	40	6	87.0	46
DENV-4	10	1	90.9	10	1	90.9	11
Total	116	4	96.7	106	14	88.3	120

## Discussion

Emerging and re-emerging infectious diseases are major burden to global public health systems (Mehand et al., 2018). Many of them are vector-borne infectious diseases that affect a large population in the tropical and subtropical regions (e.g., Africa, Latin America, and Southeast Asia), and cause higher morbidity and mortality in low-income countries than developed countries (Savic et al., 2014). Several recent outbreaks of vector-borne infectious diseases are caused by viruses, such as DENV, Zika virus, and yellow fever virus (Weaver et al., 2018). Development of point-of-care tests (POCT) for these viruses plays a crucial role in the prevention and control of vector-borne infectious diseases in resource-poor countries/regions (Kozel and Burnham-Marusich, 2017). In this study, we developed a mismatch-tolerant LAMP method and demonstrated its practical use by improving the sensitivity and specificity of a previous established RT-LAMP assay for the detection of all four DENV serotypes.

Our novel assay has notable features that are superior to the conventional LAMP method, which was developed in about two decades ago (Notomi et al., 2000). The conventional LAMP assay has been widely used in various fields because of its noted advantages including relatively independent of expensive instruments, stable reaction system, and flexibility in real-time monitoring of reactions using fluorescent dyes (e.g., SYTO 9 and SYBR green I) or visual determination using colorimetric dyes (e.g., HNB, Calcein or pH-sensitive dyes) (Tomita et al., 2008; Fischbach et al., 2015; Tanner et al., 2015). LAMP requires three pairs of primers: long FIP and BIP primers that bind to a relatively short genomic region (usually less than 300 bp); the 3′-ends of the F3/B3, FIP/BIP and FLP/BLP that provide 3′-hydroxyl groups and start DNA extension; the 5′-ends of the primers FIP and BIP (i.e., 3′-end of F1 and B1) that are responsible for the self-priming of the dumb-bell form DNA generated in the first stage of LAMP reaction (Notomi et al., 2000). However, the presence of mismatches at these ends markedly reduced the amplification efficiency of LAMP, as we have observed in this study and other have reported previously. In particular, the mismatches occurring at both ends of FIP/BIP and the 3′-ends of FLP/BLP had larger inhibition effect on LAMP amplification than those occurring at 3′-ends of F3/B3. One plausible reason for this phenomenon is that F3/B3 are only responsible for the initiation of LAMP cycling by strand replacement of newly synthesized DNA along the target, but do not participate in the self-priming of the dumb-bell form DNA (Notomi et al., 2000). Therefore, the conventional LAMP is very susceptible to mismatches between primers and templates.

High susceptibility of LAMP to mismatches limits its power in diagnostic application for viral infectious diseases (de Paz et al., 2014), and makes it especially difficult to apply on highly variable viral genome, for which the design of universal primers for a single assay that covers all genotypes, subtypes or rare variants of the same virus is challenging. Moreover, many viruses exist as quasispecies in which various variants can form mismatches with LAMP primers that designed with even the most conserved genomic region. In the last decade, a strategy by combining multiple degenerate primers together was developed for broad-spectrum detection of various genotypes or subtypes of genetically diverse viruses such as DENV, HIV-1, influenza A viruses, and enteroviruses (Poon et al., 2005; Teoh et al., 2013; Yamazaki et al., 2013; Dauner et al., 2015; Ocwieja et al., 2015; Curtis et al., 2018). However, low detection efficiency of LAMP for various viral variants still remains to be solved.

We have previously reported that by removing the mismatched bases in the 3′-end of a primer using high-fidelity DNA polymerase that has a 3′–5′ exonuclease activity, the performance of PCR for detection of genetically diverse viruses can be significantly improved (Li et al., 2019). A similar idea had been applied by others in the proof-reading PCR and the high-fidelity DNA polymerase-mediated qPCR (Bi and Stambrook, 1998; Zhang et al., 2017). In the current study, to improve the performance of LAMP in detection of genetically diverse viruses, we used similar principle and developed a mismatch-tolerant RT-LAMP method. The most distinctive feature of the novel method in comparison to the conventional method is that a minuscule amount of high-fidelity DNA polymerase was added into the standard LAMP system. In fact, only 0.15 U of high-fidelity DNA polymerase per 25 μl LAMP reaction mix is sufficient to accomplish optimal performance. As expected, the addition of high-fidelity DNA polymerase largely improved LAMP amplification efficiency for variable templates that form mismatches with the primers, thus demonstrating an excellent tolerance for various mismatches between primers and templates. Furthermore, compared to the conventional LAMP method, the mismatch-tolerant method not only significantly improved the detection sensitivity, but also markedly shortened the reaction time regardless of the presence or absence of mismatches between the primers and templates. The underlying mechanisms for an improved amplification for wild-type template have not been determined. Nevertheless, our findings indicated that the novel mismatch-tolerant RT-LAMP method is faster and more efficient than the conventional methods.

Having completed the proof-of-concept studies, we went on to test the robustness of the novel assay on clinical samples by choosing a genetically diverse virus, DENV, which causes an infection of great impact to public health in tropical and subtropical area (Bhatt et al., 2013). Early and rapid detection of DENV infection is important for patient management and epidemiological monitoring, especially in resource-limited settings (Shu and Huang, 2004; Teles et al., 2005). Current assays for dengue diagnosis have their limitations. NS1 antigen detection assay is highly sensitive and specific for acute DENV infection, but the pre-existing anti-NS1 IgG antibody induced by a previous infection will interfere with the detection of subsequent DENV infection especially in high-prevalence regions where many DENV serotypes co-circulate (Teles et al., 2005; Fuchs et al., 2014). In principle, NAA tests are more sensitive and specific for early and accurate detection of DENV infection than the NS1 antigen assay. Therefore, we used the novel method to improve a previously established RT-LAMP assay for pan-serotype detection of DENV (Teoh et al., 2013). Compared to the previous assay, the novel assay showed a significant improvement for detection of all four DENV serotypes. DENV infected patients often had mean viremia of 10^7.6-8.5^ PFU per ml of plasma at the acute phase (Vaughn et al., 2000). LOD tests revealed that the novel assay is sufficiently sensitive for detecting these viremia levels. Moreover, the evaluation of 153 clinical samples showed that the novel assay had higher detection rate than the NS1 antigen assay and RT-PCR method for all four DENV serotypes.

In resource-limited settings, diagnosis of dengue relies mainly on the clinical manifestation, which is easily confused with similar symptoms caused by other infectious agents, such as malaria, chikungunya, and influenza (Henchal and Putnak, 1990; Gubler, 1998). The novel RT-LAMP method has a high sensitivity of detection for multiple viral genotype/subtypes with a short amplification time, and can be developed into a visual measurement qualitative assay by adding HNB, cresol red, or other pH-sensitive dyes (e.g., phenol red and neutral red). The above features offer great application potential for our assay in POCT at local clinics and even in the fields. In particular, recent progresses in the nucleic acid extraction-free protocols and lyophilized formulation of LAMP reagents will further facilitate its application in the diagnosis of various viral infectious diseases in different settings in resource-poor countries (Carter et al., 2017; Liu et al., 2018).

Despite having many significant findings, this study also has several limitations. First, because there lacks a gold standard to determine the true positive and true negative samples for DENV infection, we were unable to calculate the sensitivity, specificity and accuracy of the novel DENV RT-LAMP assay. Second, the sample size used in the clinical evaluation was relatively small, especially DENV-2 samples and DENV-negative samples. Further validation of our assay with a large sample size is needed. Third, in this study, we only applied the novel RT-LAMP method to DENV detection. Its application to the detection of other genetically diverse viruses may help to further evaluate the robustness of our new assay.

## Conclusion

A novel mismatch-tolerant LAMP was developed by simply adding a minuscule amount of high-fidelity DNA polymerase in addition to the standard amount of Bst DNA polymerase to the standard LAMP reaction mixture. This method could be applied to update all LAMP-based diagnostic assays. Importantly, the novel LAMP method tolerates well mismatches between primers and templates, and has higher amplification efficiency for viral variants than the conventional LAMP method. Therefore, the mismatch-tolerant LAMP method represents a simple, sensitive and promising approach for molecular diagnosis of genetically diverse viruses, and it is especially suited for application in resource-limited settings.

## Ethics Statement

This study was approved by the Ethics Committees of Shanghai Public Health Clinical Center and Kunming University of Science and Technology. Written informed consents were obtained from all dengue-suspected patients.

## Author Contributions

CZ conceived and designed the study. YZ, SY, ML, and BW performed the experiments. CZ, YZ, ZW, YL, YH, XJ, and NY analyzed and interpreted the data. CZ, XJ, and XX contributed reagents and materials. CZ and ZW drafted the manuscript. XJ contributed to critical revision of the manuscript. CZ and NY supervised the study.

## Conflict of Interest Statement

The authors declare that the research was conducted in the absence of any commercial or financial relationships that could be construed as a potential conflict of interest.

## Footnotes

1 http://vassarstats.net/clin1.html
