# Supplementary material for: A Mismatch-Tolerant Reverse Transcription Loop-Mediated Isothermal Amplification Method and Its Application on Simultaneous Detection of All Four Serotype of Dengue Viruses
Source: Front Microbiol. 2019 May 8;10:1056. doi: 10.3389/fmicb.2019.01056 (PMC6518337; doi:10.3389/fmicb.2019.01056)
Supplement: Supplementary file 1 [file Data_Sheet_1.docx]

**A mismatch-tolerant reverse transcription loop-mediated isothermal amplification method and its application on simultaneous detection of all four serotype of dengue viruses**

Yi Zhou^1, 2 #^, Zhenzhou Wan^3 #^, Shuting Yang^4 #^, Yingxue Li^2^, Min Li^5^, Binghui Wang^4^,Yihong Hu^2^, Xueshan Xia^4^, Xia Jin^5^, Na Yu^1^*, Chiyu Zhang^2^ *

**Supplementary Information**

**Supplementary Table S1. Primers and probes used in the study.**

| **Aim** | **Primers** | **Sequence** (5’-3’) | **Ref.** |
| --- | --- | --- | --- |
| Mutant construction | F3-Mu-A(F) | CAAACCGTGCTGCCTGAAGCTCCGCCAAT | This study |
|  | F3-Mu-A(R) | TCAGGCAGCACGGTTTGAATCGTCG |  |
|  | F3-Mu-G(F) | CAAACCGTGCTGCCTGGAGCTCCGCCAAT |  |
|  | F3-Mu-G(R) | CCAGGCAGCACGGTTTGAATCGTCG |  |
|  | F3-Mu-C(F) | CAAACCGTGCTGCCTGCAGCTCCGCCAAT |  |
|  | F3-Mu-C(R) | GCAGGCAGCACGGTTTGAATCGTCG |  |
|  | F2-Mu-A(F) | CACGGAAGCTGTACGAGTGGCATATTGGA |  |
|  | F2-Mu-A(R) | TCGTACAGCTTCCGTGGCGCATGGCCTC |  |
|  | F2-Mu-T(F) | CACGGAAGCTGTACGTGTGGCATATTGGA |  |
|  | F2-Mu-T(R) | ACGTACAGCTTCCGTGGCGCATGGCCTC |  |
|  | F2-Mu-G(F) | CACGGAAGCTGTACGGGTGGCATATTGGA |  |
|  | F2-Mu-G(R) | CCGTACAGCTTCCGTGGCGCATGGCCTC |  |
|  | F1-Mu-A(F) | GGTTAGAGGAGACCCCATCCCATCACTGAC |  |
|  | F1-Mu-A(R) | TGGGGTCTCCTCTAACCGCTAGTCCA |  |
|  | F1-Mu-C(F) | GGTTAGAGGAGACCCCCTCCCATCACTGAC |  |
|  | F1-Mu-C(R) | GGGGGTCTCCTCTAACCGCTAGTCCA |  |
|  | F1-Mu-G(F) | GGTTAGAGGAGACCCCGTCCCATCACTGAC |  |
|  | F1-Mu-G(R) | CGGGGTCTCCTCTAACCGCTAGTCCA |  |
|  | BLP-Mu-G(F) | CAGAGATCCTGCTGTCTGTGCAACATCA |  |
|  | BLP-Mu-G(R) | CAGACAGCAGGATCTCTGGTCTTTCCCAG |  |
|  | BLP-Mu-A(F) | CAGAGATCCTGCTGTCTATGCAACATCA |  |
|  | BLP-Mu-A(R) | TAGACAGCAGGATCTCTGGTCTTTCCCAG |  |
|  | BLP-Mu-T(F) | CAGAGATCCTGCTGTCTTTGCAACATCA |  |
|  | BLP-Mu-T(R) | AAGACAGCAGGATCTCTGGTCTTTCCCAG |  |
| RT-LAMP assay of DENV | F3/134 | CAAACCGTGCTGCCTGT | Teoh et al., 2013 |
|  | F3/2 | TGAGTAAACTATGCAGCCTGT |  |
|  | B3/123 | ACCTGTTGATTCAACAGCACC |  |
|  | B3/4 | ACCTGTTGGATCAACAACACC |  |
|  | FIP/123 | AGGGGTCTCCTCTAACCRCTAGTCTTTCAAACCRTGGAAGCTGTACGC |  |
|  | FIP/4 | AGGGGTCTCCTCTAACCRCTAGTCTTTTTTGCCACGGAAGCTGTACGC |  |
|  | BIP/123 | ACAGCATATTGACGCTGGGARAGACGTTCTGTGCCTGGAATGATGCTG |  |
|  | BIP/4 | ACAGCATATTGACGCTGGGARAGACGCTCTGTGCCTGGATTGATGTTG |  |
|  | BLP/1234 | CAGAGATCCTGCTGTCTC |  |
| RT-qPCR assay of DENV | Pan-DENV-F | AAGGACTAGAGGTTAGAGGAGAC | [Go et al., 2016](#_ENREF_1) |
|  | Pan-DENV-R | GGCGTTCTGTGCCTGGAATGAT |  |
|  | Pan-DENV-P | FAM-CCAGAGATCCTGCTGTCTC-MGB-NFQ |  |

The artificially introduced mutations are highlighted in red.

**Supplementary Figure S1. Sequence comparison among four DENV standard strains.** Only the primer regions having mutations are shown.

| **30 minutes** | 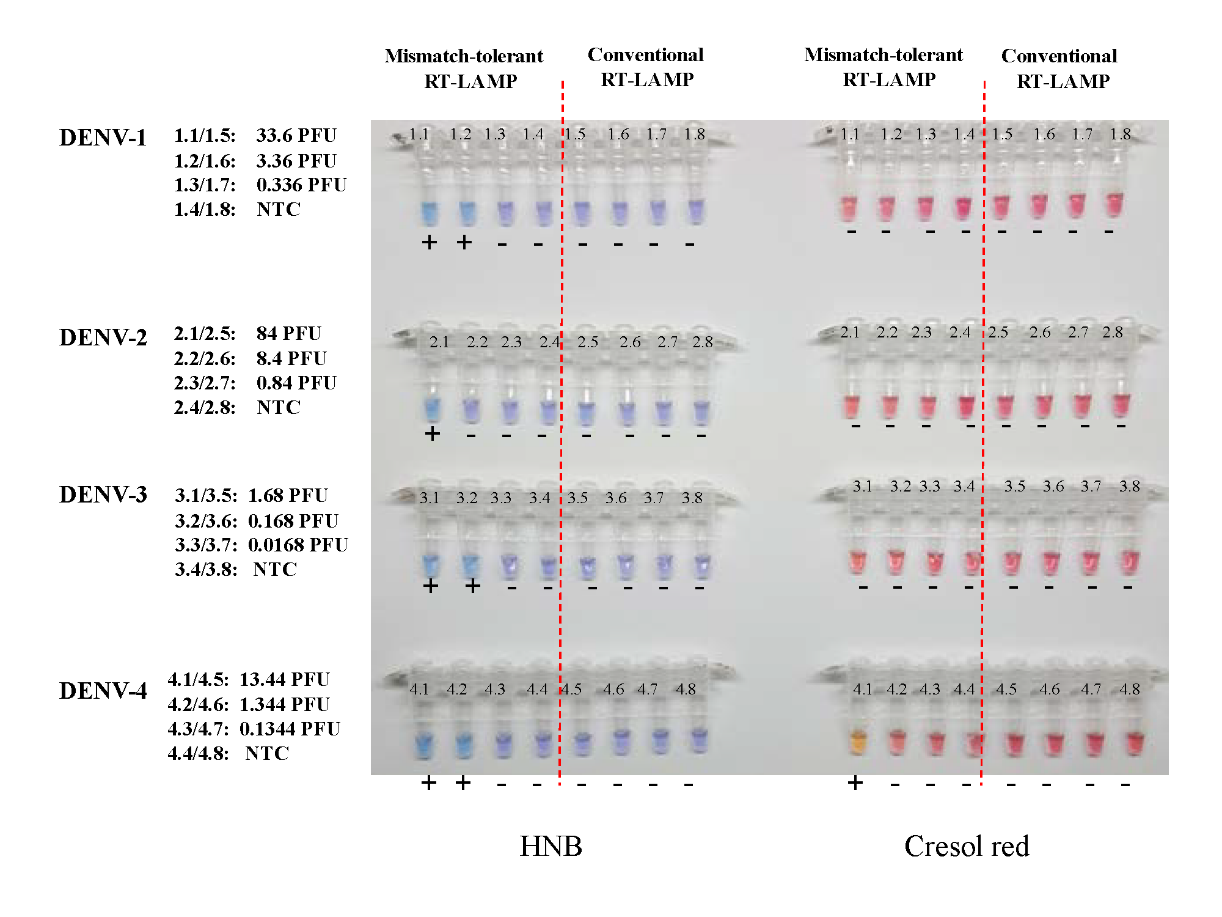 |
| --- | --- |
| **40 minutes** | 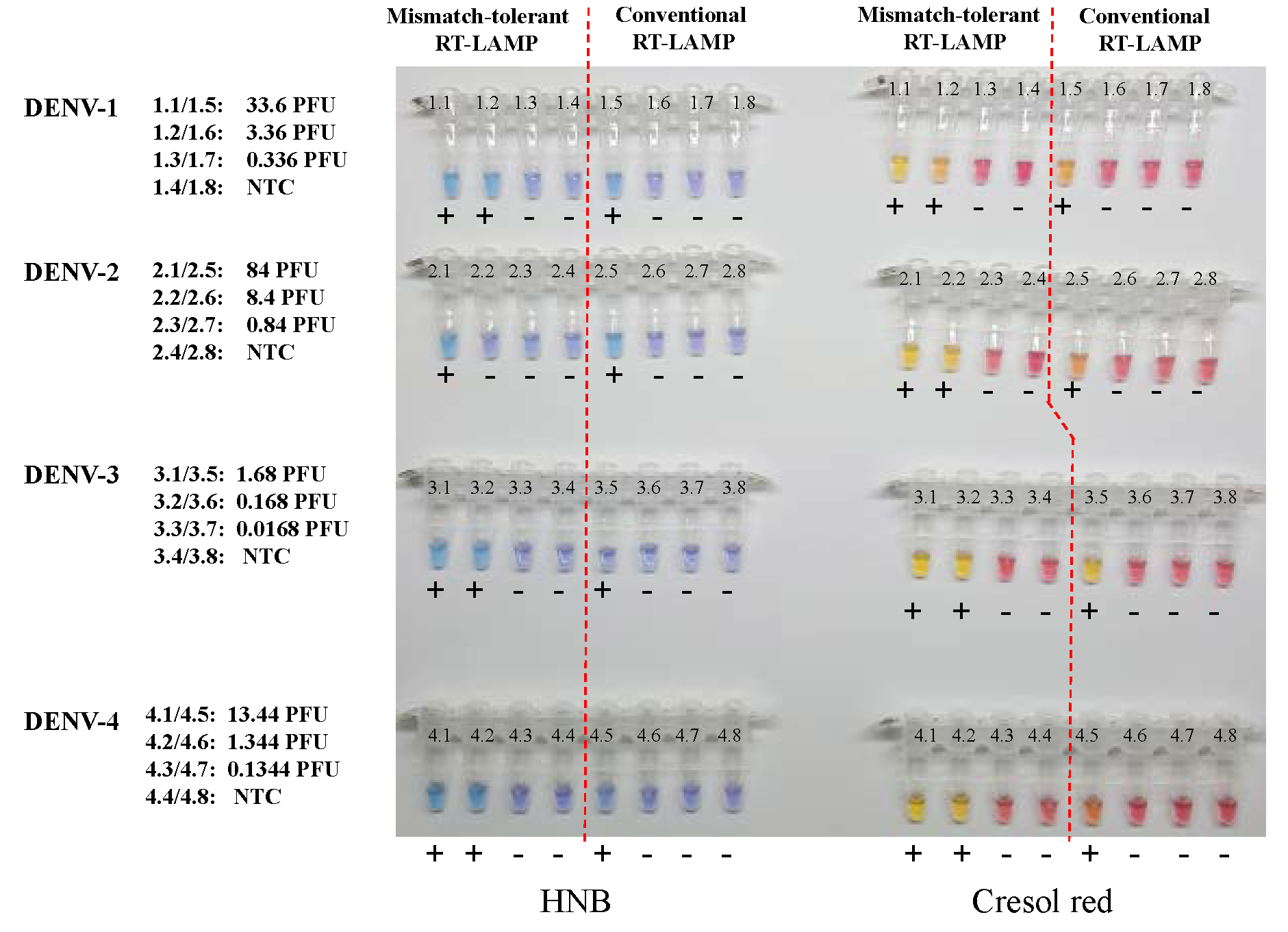 |
| **60 minutes** | 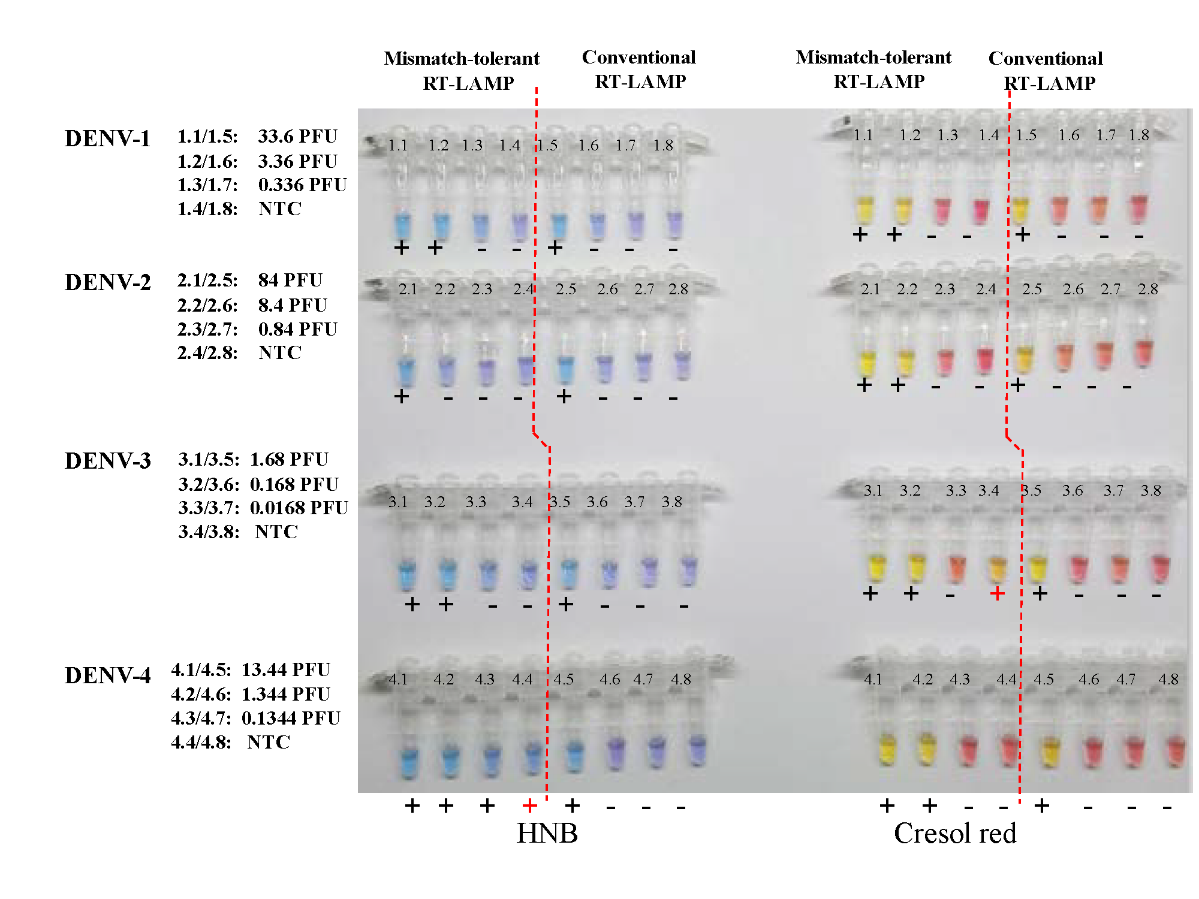 |

**Supplementary Figure S2. Visual detection of four DENV serotypes using the novel mismatch-tolerant and the conventional RT-LAMP assays at 30-, 40- and 60-minute time points.** The color changes from violet to azure for HNB and from burgundy to orange or yellow for cresol red were considered as positive (+).


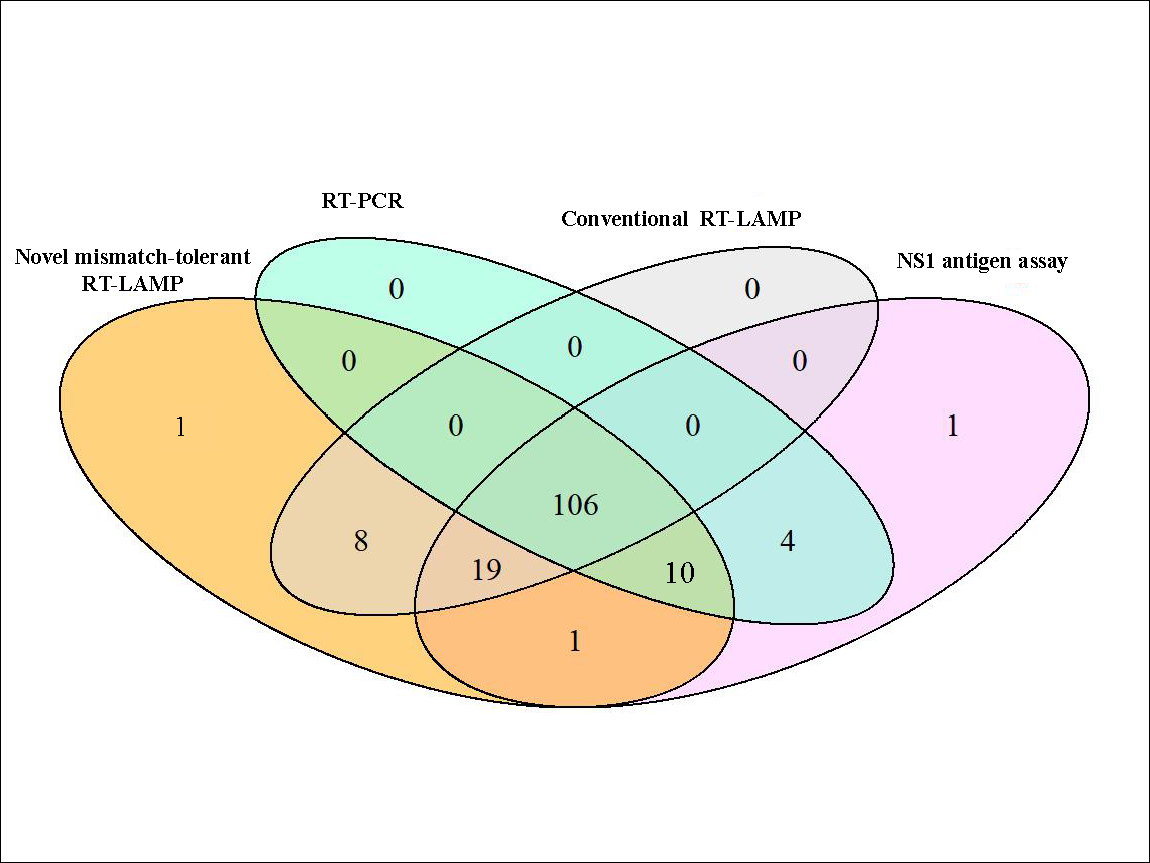


**Supplementary Figure S3. Numbers of DENV-positive samples detected by four different assays.** Because 3 samples were negative by all the four assays, the total number of samples was 150 in this Venn diagram.

**Supplementary Figure S4. Sequence analysis of the samples with a Tt difference of more than 15 minutes between the novel and the conventional RT-LAMP assays.** Only the F2 region is shown because it is where mutations were detected in some samples.
